# Curriculum Innovations: Improving Residents' Knowledge and Interest in Outpatient Neurology Through an Interactive Patient-Centered Didactic Series

**DOI:** 10.1212/NE9.0000000000200043

**Published:** 2023-02-01

**Authors:** Christopher T. Doughty, Galina Gheihman, Tracey A. Milligan, Tracey A. Cho

**Affiliations:** From the Department of Neurology (C.T.D., G.G.), Brigham and Women's Hospital/Harvard Medical School, Boston, MA; Department of Neurology (T.A.M.), New York Medical College/Westchester Medical Center, Valhalla; and Department of Neurology (T.A.C.), University of Iowa, Iowa City.

## Abstract

**Introduction and Problem Statement:**

Neurology residency training is inpatient focused, underemphasizing outpatient disorders. We implemented a novel didactic series of facilitated discussions between a patient and their outpatient neurologist to expose residents to outpatient topics and management skills.

**Objectives:**

(1) Improve residents' understanding of the roles and responsibilities of the neurologist in the outpatient setting; (2) share with residents the patient's perspective of living with chronic neurologic disease; and (3) improve residents' understanding of what effective shared decision making entails.

**Methods and Curriculum Description:**

Residents in an academic neurology program participated. Six bimonthly, 1-hour sessions were piloted in person in 2016; participants were surveyed after each session to refine the format. The formal program (6 sessions) was held virtually in 2020–2021. Each session focused on 1 disorder. The format was conversational and moderated by a course director. Discussion points were preplanned and focused on patients' experiences living with chronic neurologic disease and shared decision making. Residents, participating faculty, and patients were surveyed at the conclusion of the 2020–2021 series to evaluate its effectiveness.

**Results and Assessment Data:**

Fifty-five survey responses were completed by residents during the pilot. Only 12 residents (22%) indicated that they longitudinally followed more than 1 patient with the condition represented in the session. Qualitative comments from residents and faculty (n = 5) identified that hearing the patient perspective was the most valuable component of the series. Twenty-one of 54 residents evaluated the final program. A majority of residents, 100% of faculty (n = 6), and 100% of patients (n = 6) felt that the program's 3 learning objectives were met. Forty-eight percent of residents reported increased interest in outpatient careers. Faculty agreed that the session format was as effective as traditional lecture, without added preparation burden. Patients felt that sharing their experiences would help physicians better understand their illness and improve care for future patients; all would participate again.

**Discussion and Lessons Learned:**

Our series effectively educated residents about underrepresented outpatient topics. Hearing patients' perspectives was instrumental in achieving our learning objectives. Key factors for successful implementation included a faculty moderator, preplanned questions, and teaching slides to emphasize key learning points. Future work should evaluate whether residents' increased knowledge and interest translates into sustained behavior change and more residents selecting outpatient careers.

## Introduction and Problem Statement

The majority of neurologic care is delivered in the outpatient setting. Despite this, most neurology residency programs remain inpatient and intensive care focused.^1-3^ This leaves many trainees with insufficient knowledge about outpatient-based specialties and inadequately prepared to take on the specific roles and responsibilities required of outpatient providers, such as longitudinal management and effective shared decision making (SDM). A survey of program directors identifies a need for more outpatient training and dedicated conferences.^4^

To address this gap, we implemented a novel didactic series involving facilitated interviews between a subspecialist outpatient neurologist and one of their patients. The series format is unique in that both a subspecialty neurologist and one of their patients are present together in person to teach residents. We chose to include patients in these sessions to facilitate residents' learning about how management decisions and the impact on the patient change throughout the course of chronic neurologic conditions. This article reports on the implementation and evaluation of our series. Our results offer guidance for neurology and other training programs seeking to expand residents' exposure to outpatient topics and care.

## Objectives

We developed and implemented the Patient-Centered Outpatient Management Series. The objectives of this series are as follows:

1. To improve residents' understanding of the roles and responsibilities of the neurologist in the outpatient setting.
2. To share with residents the patient's perspective of living with chronic neurologic disease.
3. To improve residents' understanding of what effective SDM entails.

We hypothesized that the participation of patients would enhance the educational value of the series and provide an engaging approach to teaching SDM.

## Methods and Curriculum Description

### Pilot Program and Evaluation

This series started in 2016 with a pilot of six 1-hour sessions. Didactics were traditionally held in person at 1 of the 2 academic medical centers in our combined program, with residents at the other center, off-site, or at home participating via live videoconference. Didactic sessions are also attended by medical students and rotating residents from other specialties.

Each session focused on understanding the longitudinal management of a single neurologic condition for which key diagnostic and/or management decisions are made in the outpatient setting. A clinical expert in the relevant subspecialty led the session and invited a patient who they have cared for over multiple years, both to expose residents to a longer course of illness and to allow provider-patient pairs to discuss shared decisions over multiple points of their disease course. Faculty were instructed to consider patients who are articulate, knowledgeable about their illness and its course, and comfortable sharing personal details about their experience with illness. The 6 pilot sessions focused on long-term recovery after severe traumatic brain injury (TBI), amyotrophic lateral sclerosis (ALS), treatment-refractory Parkinson disease and deep brain stimulation (DBS), treatment-refractory epilepsy, genetic dystonia, and refractory chronic migraine.

Sessions were conversational between the faculty and the patient. Faculty alternated between questions for the patient and their own teaching points about managing the condition. Teaching points were agreed on with the series organizer beforehand, with a focus on practical not-in-the-textbook questions about outpatient management.

Evaluation of the pilot program aimed to evaluate the effectiveness of the series format and identify opportunities for improvement. A written survey was distributed to any residents in attendance immediately following each session. Participation in the survey was voluntary, and the only identifying information collected was postgraduate year (PGY). Residents attending multiple sessions could fill out an evaluation following each session. Participating faculty were invited to complete a voluntary online survey following their individual session. Patients were invited to participate in a voluntary 5–10-minute semistructured interview after the session. Surveys and interview questions are available in eAppendix 1 (links.lww.com/NE9/A16). For qualitative analysis, free-text responses written by residents and faculty and summarized transcripts from patient interviews were reviewed, and exploratory thematic analysis was performed.

### Final Program and Evaluation

The final program format was modified in response to the results of the pilot evaluation. Each session remained conversational between an outpatient specialist and a patient, but the series organizer (C.T.D.) now served as moderator, a written outline of questions was generated beforehand, and teaching slides were included (see Results for additional details). Strengths identified during the pilot also helped refine our stated learning objectives for the formal program. Sessions continued intermittently without evaluation in 2018–2019 to finalize the format; the formal program launched in 2020–2021.

Six 1-hour sessions were held bimonthly and focused on muscular dystrophy, multiple sclerosis, treatment-refractory epilepsy, functional neurologic disease, diagnosis and caregiver support in early-onset Alzheimer disease, and chronic migraine. Each was delivered over synchronous video conferencing due to the coronavirus disease 2019 (COVID-19) pandemic and social distancing requirements. The moderator, faculty instructor, and patient were all remote from each other and the residents.

We conducted a mixed quantitative and qualitative assessment of the final program's ability to meet our formal objectives. All adult neurology residents in our program (54 residents total, 18 per PGY) were invited to complete a single voluntary online survey at the conclusion of the academic year. We evaluated the series' 3 objectives with questions regarding residents' understanding and attitudes about outpatient management, the patient experience of illness, and SDM (Kirkpatrick level 2), using a 5-point Likert scale. We asked additional questions about residents' performance of SDM by self-report (Kirkpatrick level 3), series format, and influence on career plans. Participating faculty were invited to complete an online survey, and patients were invited to participate in a voluntary, 5–10-minute semistructured interview following their session. Both faculty and patients were asked directly to what extent the series' 3 objectives were met. Surveys and interview questions are available in eAppendix 1 (links.lww.com/NE9/A16). Qualitative content analysis was used to investigate common themes emerging among resident, faculty, and patient responses.

### Standard Protocol Approvals, Registrations, and Patient Consents

The evaluation of our pilot and final program was reviewed by the Mass General Brigham institutional review board prior to the first session and considered to be of limited risk to participants, thus exempt from full review. Patients gave verbal consent to participate in the postsession interview. We separately obtained written consent to record the session for educational purposes. All surveys were delivered via REDCap, and participants received no incentives for completion. Patient interviews were summarized in real time and not audio recorded.

### Data Availability

Anonymized data not published within this article will be made available by request from any qualified investigator.

## Results and Assessment Data

### Pilot Evaluation

#### Residents

**Figure 1:**
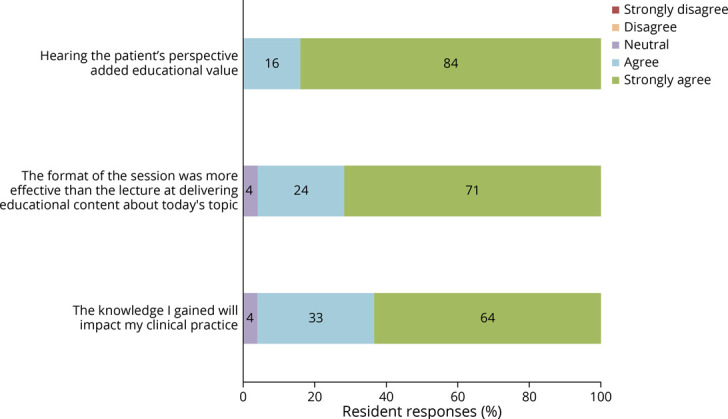
Pilot Program Resident Survey Responses Resident responses (n = 55) to survey questions distributed at the conclusion of each of 6 sessions during 2016 pilot program.

**Figure 2:**
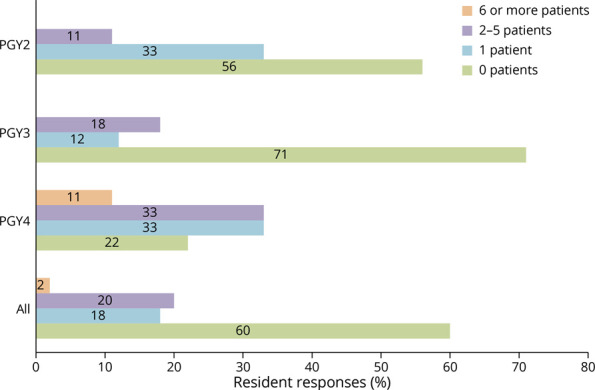
Number of Representative Outpatients Followed per Resident At the conclusion of each of the 6 sessions in our 2016 pilot series, residents were asked how many patients they had followed who had the specific condition that was the focus of the session. For example, “How many patients with migraine have you personally followed in clinic that are receiving an interventional therapy (e.g., botox, nerve blocks)?” Responses (n = 55) were aggregated across the 6 sessions and expressed as a percentage of residents responding in each category. PGY = postgraduate year.

Fifty-five postsession questionnaires were completed—3 by PGY-1 residents, 9 by PGY-2 residents, 17 by PGY-3 residents, and 9 by PGY-4 residents. All resident responses indicated that hearing the patient perspective added educational value to the conferences (Figure 1). Ninety-five percent felt that this format is more effective than lecture at delivering educational content about the topics chosen, and 97% affirmed that the knowledge gained would affect their clinical practice. Seventy-eight percent of resident respondents indicated that they had followed no more than 1 patient with the problems highlighted in these conferences during their residency, with 60% having never followed a similar patient (Figure 2). Even among the 9 PGY-4 resident responses, 5 had followed 0–1 representative patients.

Three themes emerged from residents' written comments:

1. **The central value of the patient perspective:** Residents reported that hearing directly from the patient was the most useful aspect of the sessions and described this as “fun,” “poignant,” “effective,” and a unique experience “we don't often see as…residents.” Residents appreciated hearing about problems that patients personally identified as being important to them. For example, the individual with ALS volunteered that he had thought about suicide, an uncommon topic in lectures on ALS.
2. **The session format supports learning about outpatient management:** The diseases and management issues covered in these sessions were rarely seen during residents' clinical rotations; residents felt that the series offered a “perfect format to address [the] topics chosen” and highlighted “the importance of SDM” among other topics in outpatient management.
3. **Seasoned faculty served as role models:** The perspective of seasoned outpatient clinical faculty was felt to be additive and dovetailed with the patient perspective. Faculty served as role models for communication witnessed by residents in real time. Furthermore, hearing a balanced perspective of both parties side by side informed the contributions of faculty and patients to the decision-making process.

Table 1 includes a summary of the key themes and representative quotes from residents identified in the 2016 pilot evaluation.

**Table 1:** Major Themes From Qualitative Evaluation of Pilot Program (2016–17) and Select Illustrative Quotes

**Residents**
**The central value of the patient perspective** “Important to hear directly from the patient how our treatment decisions impact people's lives, families, and emotions.” “[It was useful] to hear about how patient's lives are impacted by chronic disease—what they would have found useful to hear at the beginning.” **The session format supports learning about outpatient management** “I appreciate the question-and-answer discussion format as I think it is more interactive than a lecture and therefore more engaging.” “I found the format to be quite unique and different from other noon conferences. In general, we either see the purely didactic noon conference or the more clinical master clinician rounds (where there is less didactic content). This seminar provides both didactic and clinical components in an interactive and interesting way.” **Seasoned faculty served as role models** “The combo of the patient's story and expert opinion is invaluable.” “One aspect that is difficult to appreciate in typical didactic sessions is how patients manage with empiric trial-and-error management of headaches; it was great hearing…both the patient and doctor perspectives.”
**Faculty**
**Strengthening patient-clinician relationship** “I think it was helpful for everyone involved. It brought the patient and I closer and to another level that will be helpful in care. I think it was helpful for the residents. And I know the patient enjoyed taking part.” “[The most useful aspect for me] was probably strengthening my relationship with the patient and, I hope, making her feel more empowered.” **Increased understanding of common outpatient disorders** “For the residents, I would hope that they realize that all “functional” patients have brain conditions, even if we don't know how to diagnose or treat them—and that having that attitude towards a patient … decreases the patient's shame, or feeling responsible for their symptoms.”
**Patients**
**Helping physicians learn about and value the patient perspective** “I agreed to do it because I think the people that are going to be involved in the patient's care should be aware of what the patient experiences. The more they know about the real-life experience, the better prepared they will be.” “I like the idea of taking patients' perspective into consideration. It is important for doctors to hear from people of different backgrounds. Sometimes, doctors will ask me a question and I think they don't realize how my background affects my knowledge and experience.” **Participating in the sessions helped patients learn something new** “I already knew what [my neurologist] was thinking regarding my case, but there were things she said to the other doctors that made me think about it a different way. I found this very enlightening. When she put my case into a broader context…that was an “Aha” moment for me.” **Sharing and teaching is a way to “give back”** “It is great to hear that what we went through as a family is paying off and helping others.”

#### Faculty

Five of 6 faculty completed the survey. All faculty enjoyed leading the sessions and found the sessions to be easier and less time consuming to prepare for than lecture. All agreed that the format was more effective than lecture at delivering educational content about the chosen topic. All felt that having the patient present was essential to the success of the conference.

Qualitative responses highlighted faculty's appreciation of the opportunity to discuss practical, patient-centered issues distinct from book knowledge topics. Faculty also felt that the session strengthened their relationship with their patient. Key themes are summarized in Table 1.

#### Patients

Postsession interviews were completed for all 6 sessions—5 patients and the father/caregiver of the patient with TBI. All respondents would participate again, and no negative experiences were reported. Patients were motivated to share their perspective of living with their condition in hopes that this would benefit other patients. Respondents felt that the course of illness was accurately portrayed, and all were satisfied with the balance between their own participation and the participation of their physician. Benefits to participation included the satisfaction of seeing many trainees and disciplines interested in learning about their condition and gaining additional perspective on their illness and medical decision making. Key themes are summarized in Table 1.

### Programmatic Improvements in Response to Pilot

Residents, faculty, and patients were asked to identify challenges and suggestions for improvement after the pilot program. Residents expressed a desire for specific didactic instruction during key points of the conversation and for specific teaching points to be called out. Residents and patients both desired more questions and direct participation from the audience. Multiple patients commented that the interaction with the other hospital over video conferencing was unusual, as it was difficult for patients to appraise the engagement of those watching. Some residents also expressed displeasure with watching an in-person conversation over video conference. Multiple faculty participants reported that it was difficult to weave substantive clinical content naturally into the patient conversation and found it challenging to balance how much they were speaking with how much their patient was speaking.

In response to these suggestions, we established a structured format using a single moderator for all sessions (Table 2). The moderator developed an outline of preplanned questions for review by the faculty and patient before each session to solidify desired learning points, to allow better preparation, and to ensure that time was divided between the faculty and patient equitably. Sample outlines are available in eAppendix 2 (links.lww.com/NE9/A17). During the session, the moderator led the faculty and patient through the preplanned questions. Faculty were now also asked to prepare ∼5 slides to serve as a visual aid during relevant parts of the conversation, corresponding to the key learning points identified. Finally, the last 5 minutes was set aside for resident questions. Physical examination was no longer performed in the sessions, in part due to the virtual format but primarily because it was not felt to be as important for achieving the series' formal learning objectives.

**Table 2:** Key Tips for Success When Implementing This Series

1. **Choose appropriate topics:** focus on management topics within outpatient neurology not frequently encountered by residents, e.g., usually managed in subspecialty clinics or requiring multiple years of follow-up.
2. **Choose appropriate faculty members:** select an experienced outpatient neurologist and prioritize clinical expertise over research credentials
3. **Choose appropriate patients:** select a patient (a) with a prolonged disease course requiring shared decision making at multiple stages, (b) who has a good relationship with their neurologist, (c) who has a good understanding of their illness, (d) is an effective communicator, and (e) who is comfortable sharing personal details about their values and the impact of their illness. Attend to the diversity of patients selected to ensure that representative voices are heard.
4. **Plan the conversation:** identify session learning objectives and create a session guide including a summary of the patient's clinical course and anticipated questions for the neurologist and the patient. Share this guide with the patient and faculty ahead of time to help them prepare and make changes. Although each session varies, a general outline might include the following:
• Brief history from the patient's perspective of initial symptoms and signs
• Discussion of early conversations about diagnosis and impact on the patient
• Identifying key treatment decision points and sharing the patient's and the clinician's perspective
• Identifying challenges the patient may have faced during their course
• Clinician's overview for outpatient management of the featured condition
• Patient's perspective on living with their neurologic condition
5. **Moderate the session:** use a moderator during the session to manage time, direct the flow of the discussion, and balance the perspectives of the patient and the faculty member.
6. **Emphasize desired learning objectives:** ask faculty to prepare 3–5 teaching slides to illustrate key concepts.
7. **Encourage resident participation:** save time for questions.

### Final Program Evaluation

#### Residents

Twenty-one of 54 residents completed the survey (response rate 39%), with 9 PGY-2 (42% of respondents), 6 PGY-3 (29%), and 6 PGY-4 (29%) respondents. Residents attended on average 2.86 ± 1.15 of the 6 sessions; 7 residents attended 4 or 5 sessions, and zero residents attended all 6.

**Figure 3:**
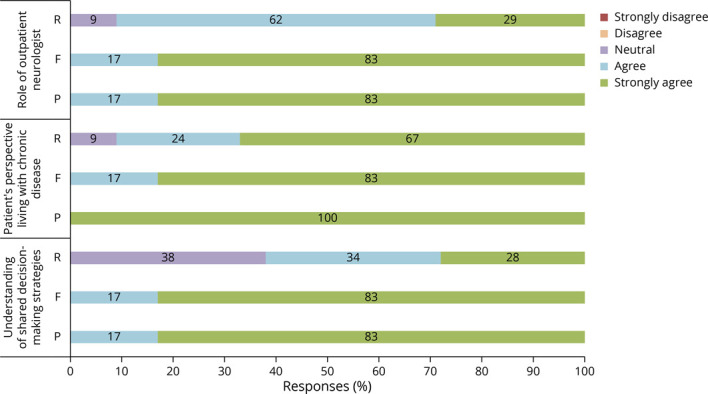
Program Success at Achieving Educational Objectives Resident (R) (n = 21), faculty (F) (n = 6), and patient (P) (n = 6) perspectives on to what extent the outpatient series met its educational objectives of (a) improving residents' understanding of the roles and responsibilities of an outpatient neurologist; (b) sharing the patient's perspective on living with chronic neurologic disease; and (c) exposing residents to effective strategies for shared decision making.

**Figure 4:**
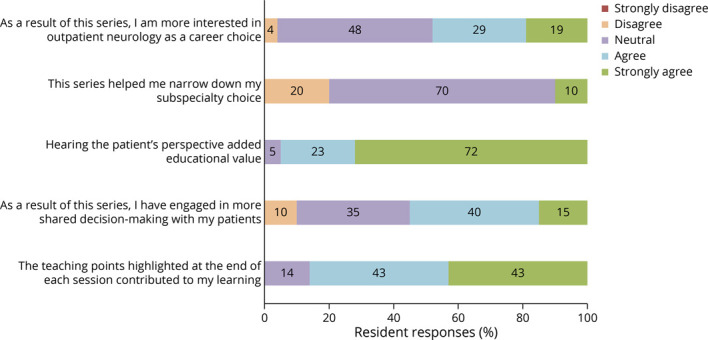
Additional Resident Survey Responses Resident responses (n = 21) to additional survey questions about session learning topics and format for the final 2020 program.

Resident responses regarding our series objectives are summarized in Figure 3. Ninety-one percent of residents agreed that they have a better understanding of the role of a neurologist in the outpatient setting as a result of this series. Ninety-one percent felt that they had a better understanding of patients' experiences living with chronic neurologic disease. Sixty-two percent agreed that they have a better understanding of what SDM entails. Additional responses are summarized in Figure 4. Notably, 95% of residents agreed the patients' perspectives added educational value. Fifty-five percent indicated that they have engaged in more SDM as a result of the series. Although only 10% reported that the series helped them narrow down their subspecialty choice, nearly half (48%) of residents indicated that the series increased their interest in outpatient neurology as a career.

Qualitative comments again indicated that residents enjoyed the session format and the opportunity to hear from both patients and their outpatient providers. The most commonly identified strength of the program remained the opportunity to learn from patients' perspectives. Table 3 describes the major themes identified from the formal program evaluation.

**Table 3:** Major Themes From Qualitative Evaluation of Final Program (2020–21) and Select Illustrative Quotes

**Focus on patient perspective and experience is highly valuable**
“[What I liked best was] hearing the patient's perspective on how their diagnosis affects their life and the factors that go into their decisions (e.g., whether to get surgery, medications, etc.).”—Resident “Great to hear about specifically about patient worries, hopes and values as it pertained to the role of epilepsy surgery.”—Resident “I think it is really important to get the patient's perspective but also to see a patient who is doing really quite well. I think for whatever reason MS gets kind of [negative reputation] … I want to give a strong voice and accurate information to the next crop of physicians/neurologists.”—Patient
**Series format is unique and effective**
“[It] is more engaging because we are hearing firsthand from the patient and their neurologist.”—Resident “The format [worked well] with practitioner and patient with a moderator to help facilitate. It's good to have pre-emptive slides that cover major topics or themes that are expected to be covered during the session.”—Resident “One thing I thought worked really well was that there were 3 voices: myself, the patient and [the moderator]. This helped to keep things engaging and interesting for the residents.”—Faculty
**Increased understanding of outpatient topics not covered elsewhere**
“We have precious little [teaching] in outpatient neurology in a program largely pressed into service of inpatient care … this series stands out as one of the few to explore and demonstrate management of complex and chronic neurological care.”—Resident “Helpful to frame the world of outpatient neurology, which is most of the field.”—Resident “I think exposure to common outpatient scenarios…is really important and helpful, to expose young neurologists to what is out there. Residents do not see a lot of the common outpatient conditions.”—Faculty
**Interaction between the physician and the patient offers insight into shared decision making**
“I learned that patients have a lot to offer in their expertise with the disease.”—Resident “Implicitly, I think they might learn that outpatient management is a long-term relationship that involves both diagnostic and management shared decisions.”—Faculty “The patient matters. It will take time between the neurologist and the patient; a lot of working together to find the best solution.”—Patient
**Modeling and instilling values of humanism**
“Refreshing and wonderful connection to outpatient neurology and the patient experience. … Really helped to instill the humanistic component of practicing neurology into our learning.”—Resident “The extreme anxiety with an unpredictable paroxysmal disorder that really requires a doctor as mentor so patient doesn't feel as scared…You can't lecture on that.”—Faculty “On a personal level, I think how you present life-changing news to patients is vitally important. If somebody remembers what I experienced in a bad moment when they are being told they have 10 things to do but need to go tell this patient a tough diagnosis, maybe they will remember to think about it, take a moment, turn off their pager, and…recognize how impactful the conversation will be.”—Patient “I wish they could be more positive with their patients. It is brutal to hear ‘we don't know if you will get better.’ I hope that they say that there are ways that you can better, that there is support for you.”—Patient “You have to believe your patient. That is the hardest part. A lot of people do not know about this diagnosis of [FND], but now I hope they know. You don't just want to say ‘you're stressed out.’”—Patient
**Unique benefits for patients: understanding, contribution, and hope**
*Understanding* “To sit down and see the slides on the screen and hear [my doctor] talk about [my diagnosis] was very helpful for me. I liked hearing it again…it helped me understand my condition a little bit better.”—Patient “I was also just reminded about the progress I have made. I didn't know that PT was such a scarce resource for people with FND, that makes me so sad. I want to be part of the solution to help address this problem!”—Patient *Contribution* “I loved that I could share my truth. The residents and my outpatient doctor told me that they were impacted by this, which felt so great…It made me feel like [the residents] really heard me.”—Patient *Hope* “I learned my doctor cares if I meet my dreams in life, that made me cry. He is working to make this happen. I knew doctors care, but it was unbelievable to learn that my doctor cares this much.”—Patient

Desired topics for the future included neuro-oncology, use of DBS in Parkinson disease, ALS, sleep medicine, autonomic dysfunction and/or small fiber neuropathy, chronic pain syndromes, and neurorecovery after stroke or TBI. One resident expressed a preference that these sessions be conducted in person.

#### Faculty

All 6 faculty completed the survey. All agreed that the sessions met the 3 main objectives (Figure 3). All felt that having a patient present was essential to the session's success. Five reported spending less time preparing for the session compared with lecture, with one spending the same time.

In qualitative comments (Table 3), all faculty endorsed learning something new from their patient. They felt that the series allowed residents to understand the patients' perspective on illness and to appreciate the importance of the patient-clinician relationship for supporting SDM with patients. Both the preplanned outline of questions and having a moderator were highly valued. One faculty participant expressed a preference that these sessions be conducted in person.

#### Patients

Postsession interviews were completed for all 6 sessions—5 patients and the husband/caregiver for the patient with dementia. All participants agreed that the sessions met the 3 main objectives (Figure 3). All stated that they would participate again. None reported negative experiences, although the caregiver for the patient with dementia reported: “The only thing that made me uncomfortable is when [the patient] got more uncomfortable. She lives in the moment and she goes to her standard fallback responses.”

Qualitative responses indicated that patients found the format welcoming and comfortable. They appreciated having a moderator and an outline of questions to review ahead of time. Patients found it enjoyable and informative to review their case history; some found more clarity on their medical decisions after hearing about their medical journey a second time. They appreciated that this series emphasized the importance of SDM to reach informed, personalized decisions. Patients were motivated to participate to help others with their condition. One patient shared: “When we first met [my doctor], we made it very clear that any way we could to help out other people, we would do it, no matter how small. We want to expose people to this condition. We don't hide from it.” Patients modeled elements of good communication and moral standards in their comments (Table 3). We also found several unique benefits of participation for patients, including improved understanding, contribution, and hope.

## Discussion and Lessons Learned

We implemented a novel, discussion-based, patient-centered outpatient neurology series that successfully educated residents about (1) outpatient disorders underrepresented in neurology training and the role of the outpatient neurologist, (2) the patient's perspective on living with chronic neurologic illness, and (3) strategies for outpatient SDM. The informal discussion-based format was preferred to and more engaging than traditional lectures, without requiring additional preparation time of faculty. Hearing patients' perspectives was considered essential and the most educational component by residents and faculty, and patients appreciated the opportunity to share their stories while learning more about their condition.

The structure of neurology residency training overemphasizes acute presentations of uncommon neurologic disorders and underemphasizes common disorders managed in the outpatient setting.^1,2^ Nearly half (46%) of adult neurology residents report insufficient outpatient neurology exposure prior to making decisions about what fellowship to pursue.^5^ A survey of program directors (1994) suggested that residents should spend more time in outpatient neurology and that an ideal training program would include conferences specifically dedicated to outpatient management issues.^4^ An important implication of the success of our series is that discussion-based didactics may be an effective way to expose residents to outpatient neurology when an increase in clinical exposure is not feasible. Most of our residents reported a better understanding of the role of an outpatient neurologist, and nearly half reported increased interest in an outpatient career as a result of the series. These conferences alone are likely not sufficient to guide residents toward outpatient careers, as only 10% reported the series helped them narrow down their subspecialty choice. We also did not assess whether an increased number of graduating residents went on to actually choose outpatient careers. However, the addition of conferences like these into residents' didactic curriculum can serve as a concrete step to augment their experience with outpatient neurology. Furthermore, by choosing faculty participants based on their clinical and teaching expertise rather than research experience, our series also elevated clinician educators and those with outpatient careers as academic role models for residents.

Our series featured live patients partnering with their outpatient neurologists to teach about their lived experiences. Patients have been used previously in medical education interventions, with both learners and patients generally evaluating such programs positively.^6-8^ However, the specific use of neurologic outpatients has not been well studied prior to our work. Previous research emphasizes the importance of selecting suitable patients: individuals who are articulate, not anxious, possess a good understanding of their illness, and able to cope if unpleasant issues are discussed.^6^ Selecting patients from diverse backgrounds can help ensure that a broad range of patient preferences and experiences are heard by residents. Challenges may arise when selecting patients with neurologic conditions, as a patient's memory, personality, or ability to communicate may be affected. During our session on early-onset Alzheimer disease, for example, our patient did not play as active a role in the discussion as her primary caregiver. However, this was true to the nature of this disease and illustrated the central role of caregivers in chronic, progressive neurologic illness. Feedback from our patients indicated that these sessions were neither a burden nor exploitative. Rather, this experience offered patients benefits similar to that reported previously,^9,10^ including the opportunity to educate future providers, to help other patients with related conditions, and to learn more about medical decision making in their specific case.

We hoped that our series would increase residents' understanding about strategies for effective SDM. There are several reasons neurology residents may lack opportunities to practice SDM. SDM is less commonly used in the inpatient setting,^11^ both because patients may be too ill to fully participate and because providers think SDM will take too much time. Residents are also more likely than practicing medical specialists to report a preference for paternalistic decision making when compared with SDM.^12^ Lack of sufficient medical knowledge about outpatient disorders (e.g., prognosis, natural history, and treatment options) may serve as a further barrier to SDM.^13^ The format of our series is well suited to educate residents about SDM, as successful SDM requires bridging the expertise of the patient (their preferences, values, and lived experiences) with the expertise of the physician (options for treatment and the medical evidence behind each). Having both patient and neurologist present in the session allows residents to hear both perspectives and witness SDM recapitulated in real time.

Despite this, our results suggest that more work may be required to adequately train residents on SDM. Although most residents did report increased understanding of what SDM entails, fewer agreed that this objective was achieved compared with our other 2 objectives. Furthermore, only 55% reported engaging in more SDM in practice. Ultimately, SDM is a skill that requires deliberate practice to master; we hypothesize that fewer residents reported this objective being met as our series did not offer residents an opportunity to practice this skill themselves. Perhaps pairing our series, which offers specific knowledge about SDM, with hands-on sessions that offer residents an opportunity to practice SDM skills^14,15^ would better achieve the aim of increasing residents' use of SDM.

Our series highlighted communication skills that residents must master to become expert outpatient neurologists. Patient-centered care is often idealized in medical education but is typically defined and taught by physicians.^16,17^ Our series allowed patients to articulate best practices for patient-centered care themselves, highlighting the most effective physician behaviors and communication skills. Our patients hoped that sharing their experiences would teach residents effective means to deliver difficult news, explain diagnoses, and/or instill hope. Positive role modeling by doctors is important in the professionalization of medical trainees^18^ and can promote learners' development of communication and character skills.^19^ Less is known about whether patients may serve as a kind of moral exemplar for character development.^20^ Patient-led seminar series like ours may fill a void in trainee education and professional development by focusing on emotional, communication, and moral issues.^21^

Our series reaffirmed the need for and importance of augmenting education in outpatient neurology and offered several lessons learned. The topics we chose—while common conditions in outpatient practice—were indeed underrepresented in our residents' training, with only one-fifth of residents following more than 1 patient with a similar condition in their own clinic. The format of our series was essential to its success, as the most beneficial aspect of the series was the ability to hear the patients' perspective directly. Feedback obtained from our pilot series allowed us to refine the session format; to aid those planning to implement a similar series elsewhere, we summarize the best practices we developed in Table 2. Key practices included having a moderator and preparing an outline of questions for patients and faculty beforehand. This ensured that patients were comfortable with the planned discussion topics (we could confirm, for example, that a patient was willing to discuss end-of-life planning) and naturally helped balance the patient and faculty perspectives ahead of time. Our transition to a virtual format as a result of the COVID-19 pandemic did not diminish the educational value of the series and was convenient for patients, faculty, and residents.

A limitation of our findings is that our series was implemented in a single residency program in an urban, academic medical setting. Local implementation and assessment of similar programs elsewhere is necessary to ensure that it meets the needs of residents in other programs. Another limitation is that each session is unique to the featured patient and provider and thus difficult to replicate. Cross-institutional sessions could be trialed to broaden the reach of this program, while leveraging the success of the virtual format. This may be an important option for programs without access to outpatient faculty in certain subspecialties. Nevertheless, we have identified key programmatic elements that can facilitate independent adaptation elsewhere. The success of the program will ultimately rest on knowledgeable faculty, articulate patients, and a well-practiced moderator.

It is possible that responder bias may have influenced our results, as residents already interested in outpatient neurology may have been likely to respond to our survey. Self-report of behavioral change is further prone to recall and social desirability biases. Future work is needed to determine whether residents' self-rated increase in knowledge translates into behavior change. Finally, we hope that the success of this program may encourage neurology educators to consider how else to incorporate patients into curricula more broadly. At minimum, in future sessions, we plan to allow patients to take a more direct role in identifying desired learning objectives for these sessions before the moderator generates an outline. We can also imagine patients playing a role in teaching or evaluating communication skills or giving feedback on SDM in a standardized OSCE.

We implemented a feasible, low-resource, and impactful educational series highlighting outpatient neurology disease management and patient-centered SDM. Our session met important objectives not currently addressed in most inpatient-heavy neurology residencies and was of interest and benefit to neurology trainees, faculty, and patients alike. Future work includes evaluating the long-term effect of this series on residents, including their understanding of SDM, the role of the outpatient neurologist, and career interests in outpatient neurology.

## Appendix Authors

**Table:**

Name	Location	Contribution
Christopher T. Doughty, MD	Department of Neurology, Brigham and Women's Hospital/Harvard Medical School, Boston, MA	Drafting/revision of the manuscript for content, including medical writing for content; major role in the acquisition of data; study concept or design; and analysis or interpretation of data
Galina Gheihman, MD	Department of Neurology, Brigham and Women's Hospital/Harvard Medical School, Boston, MA	Drafting/revision of the manuscript for content, including medical writing for content; major role in the acquisition of data; study concept or design; analysis or interpretation of data; and additional contributions: co–first author
Tracey A. Milligan, MD	Department of Neurology, New York Medical College/Westchester Medical Center, Valhalla, NY	Drafting/revision of the manuscript for content, including medical writing for content, and study concept or design
Tracey A. Cho, MD	Department of Neurology, University of Iowa, Iowa City	Drafting/revision of the manuscript for content, including medical writing for content; study concept or design; and analysis or interpretation of data

## Glossary

ALS: amyotrophic lateral sclerosis
COVID-19: coronavirus disease 2019
DBS: deep brain stimulation
PGY: postgraduate year
SDM: shared decision making
TBI: traumatic brain injury
